# Targeting the Src N-terminal regulatory element in cancer

**DOI:** 10.18632/oncotarget.28434

**Published:** 2023-05-19

**Authors:** Betlem Mezquita, Marjorie Reyes-Farias, Miquel Pons

**Affiliations:** ^1^Biomolecular NMR Lab, Departament de Química Inorgànica i Orgànica, Universitat de Barcelona (UB), Barcelona 08028, Spain; ^2^Departament de Ciències Bàsiques, Universitat Internacional de Catalunya (UIC), Sant Cugat del Vallès 08195, Spain

**Keywords:** Src family kinases, intrinsically disordered proteins, SNRE, cell-type selective drugs, drug resistance

## Abstract

The signaling pathways displayed by cancer cells are often composed by the same components than the physiological ones, yet the overall result is a pathological deregulation. The non-receptor protein tyrosine kinase Src is a good example. Src is the first described proto-oncogene and a demonstrated player in cancer progression, as it affects proliferation, invasion, survival, cancer stemness, and drug resistance. Src activation is linked to poor prognosis in many cancer types, yet mutations in this protein are rarely observed. In addition, being a demonstrated cancer target, unspecific inhibition of the kinase activity has proven inefficient in clinics since the inhibition of Src in non-cancerous cells results in unacceptable toxicity. Thus, there is a need for new target regions in Src that could inhibit Src activity only in certain cell types, e.g., cancer cells, while maintaining the normal physiological activity in healthy cells.

The Src N-terminal regulatory element (SNRE) includes the poorly studied intrinsically disordered region with unique sequences for each of the members of the Src family. In this perspective, we discuss the non-canonical regulatory mechanisms involving the SNRE and their potential use as oncotargets.

## INTRODUCTION

Src, the first discovered oncogene, is the leading member of the Src family of kinases (SFK) that includes Fyn, Yes, Blk, Yrk, Fgr, Hck, Lck, and Lyn. They are non-receptor protein tyrosine kinases transducing signals from the external environment to intracellular pathways essential for normal cell homeostasis [1, 2]. The physiological functions regulated by Src include cell proliferation and survival, cell shape, cell adhesion to other cells and the matrix, and migration. In addition, in a cancer context, Src contributes to invasion, angiogenesis, survival of metastatic cells, metabolic reprogramming [3], regulation of the inflammatory response [4], and acquisition of resistance to chemotherapy [5–9].

**Figure 1:**
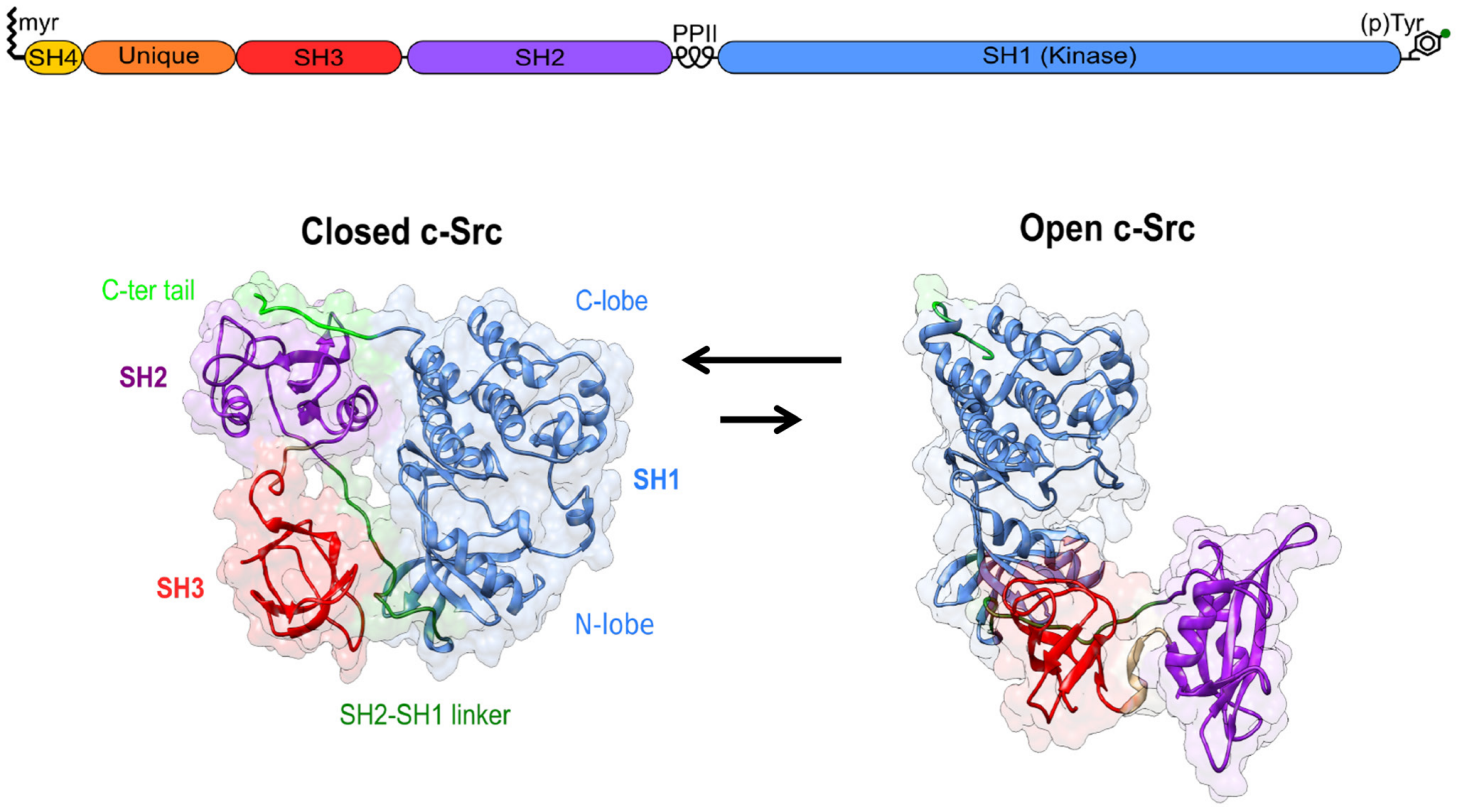
Canonical Src regulation involves the transition from an inactive closed state to an active open form. The role of the Unique disordered domain and the SH4 domain, beyond membrane anchoring, is just starting to be understood.

All SFK share a common domain structure with an N-terminal membrane anchoring SH4 domain, and an SH3 and an SH2 domain that bind proline-rich sequences and phosphotyrosine, respectively, as well as the kinase, or SH1 domain. At the C-terminal end, a conserved tyrosine residue provides a regulatory site that, when phosphorylated, engages in an intramolecular contact with the SH2 domain and contributes to stabilizing a closed inactive state. The closed state is further stabilized by the interaction of the SH3 domain with a proline-rich region in the linker connecting the SH2 and SH1 domains (Figure 1).

The SH4 and SH3 domains are separated by a long intrinsically disordered region known as the Unique domain. In contrast, to the other domains, it shows very low sequence homology among the distinct SFK members. The disordered regions, including the SH4 domain, and the SH3 domain form the Src N-terminal regulatory element (SNRE), whose mechanism is far less understood than the canonical regulation involving the SH3 and SH2 domains and tyrosine 530 in the C-terminal tail.

The canonical regulatory mechanisms are based on the displacement of the intramolecular inhibitory interactions. This can occur through dephosphorylation of tyrosine 530 or by its displacement from the SH2 domain by interaction with a phosphotyrosine residue from another molecule with a higher affinity than the intramolecular interaction. Similarly, the inhibitory interaction involving the SH3 domain can be replaced by an intermolecular interaction with another proline-rich sequence. Src activation is maintained by the phosphorylation of a tyrosine residue in a regulatory loop by a second Src molecule. The activation of Src by displacement of the intramolecular interactions results in the formation of intermolecular complexes, that can act as scaffolds recruiting other substrates or may result in phosphorylation of additional tyrosine residues, leading to a positive feedback loop.

The canonical Src signaling axes are initiated by EGFR and other receptor tyrosine kinases or integrins. For example, clustering of integrins results in transient FAK dimerization and autophosphorylation that creates high affinity binding sites for the Src SH2 domain, which causes Src recruiting, activation, and further phosphorylation of other FAK tyrosine residues and the formation of a stable complex that recruits other FAK-associated Src substrates [10]. FAK binding involves also SH3 domain binding to tandem docking sites [11]. Activation of the Src-family tyrosine kinase Hck by HIV Nef also implies the displacement of the SH3 domain [12].

In this perspective article, we focus on “non canonical” regulation involving the SNRE, although the distinction of individual regulation mechanisms is rather academic since Src is a functional unit in which the individual regions or domains act cooperatively. Interestingly, the origin of the unbalance in our understanding of the roles of the globular and disordered domains is both technical and conceptual. Disordered regions are technically more difficult to study than globular domains as they resist analysis by X-ray crystallography. Fortunately, the continuous advances in NMR have alleviated this technical difficulty. Cryo-electron microscopy cannot provide detailed information on the disordered regions, but integration with NMR is a promising strategy [13]. The more difficult challenge is the (wrong) conceptual perception that a well-folded structure is a sine-qua-non requisite for function. This perception was based on the extrapolation to the entire proteome of the observation that many folded proteins lose their activity upon denaturation, disregarding the fact that about one-third of eukaryotic proteins are intrinsically disordered and another third have long disordered regions together with folded domains [14], as in the case of Src and other SFKs.

### Non-canonical regulatory mechanisms. The role of the Unique domain

While the regulatory roles of the SH2 and SH3 domains in Src family kinases are relatively well understood, the function of the intrinsically disordered Unique domain is often unappreciated.

In a series of publications our group showed that mutations introduced in a region defined based on NMR observations [15] resulted in significant changes in physiological processes in which c-Src was involved. This region was located next to the singular ^64^FGG^66^ motif that is also found in the related Src family kinases Fyn and Yes. We named it as “Unique Lipid Binding Region” (ULBR) because the first *in vitro* measurements showed that one of the effects of mutations in this region was a decrease in its capacity to interact with lipids [16]. Initial effects were detected in the progesterone-induced Xenopus laevis oocytes’ maturation, which requires Src. Expression of ULBR Src mutants resulted in similar maturation rates as in the controls expressing wild-type Src but, once matured, a significant fraction of the oocytes expressing mutant Src died, in contrast to the ones expressing wild-type Src. This was the first indication that the Unique domain has a regulatory function and that its effect is cell-type sensitive [16]. ULBR mutants of human Src were tested by expressing the mutants in NIH3T3 and human colorectal cancer cell lines overexpressing either wild type or ULBR mutants. The ULBR mutants of Src caused a reduced transforming capacity as compared to wild type Src and showed reduced tumor development in nude mice [17]. However, the ULBR mutants did not significantly alter the global tyrosine phosphoproteome in colorectal cells, suggesting that the effect of the ULBR mutations is not a direct inhibition of the kinase activity but has an impact on the capacity to phosphorylate specific substrates needed for oncological signaling [17].

At the atomic level, the Unique domain is an intrinsically disordered region (IDR). This means that it does not adopt a single well-defined three-dimensional structure, even in its native form [18]. IDRs are very abundant in eukaryotes but rarely observed in prokaryotes [19]. IDR are most often associated with high-level regulation, and is not surprising that they are very commonly found in proteins whose deregulation results in cancer, cardiovascular or neurodegenerative diseases [20]. Despite remaining disordered, the Unique and SH4 domains form a fuzzy intramolecular complex around the SH3 domain [21, 22]. Fuzzy intermolecular complexes were introduced by Fuxreiter and Tompa [23] and are often the manifestation of the coexistence of multiple weak complexes in rapid exchange that modulate the conformational ensemble of the disordered region while still allowing a very large plasticity and capacity to respond to its environment [24]. The intramolecular version of fuzzy complexes refers to the interaction of disordered regions with a neighbor folded domain, which in the case of Src is the SH3 domain. An analysis of disordered linker regions in human proteins suggests that the SH3 domains may often be nucleating intramolecular complexes. Thus, the situation found in Src may be much more general [25]. The plasticity of the disordered regions may have a special role in the context of cell signaling as “environmental readers” because the weak interactions shaping their conformational space makes them especially sensitive to even small changes in their environment and enable the integration of multiple input signals, making them “molecular computers”. In this context, intramolecular fuzzy complexes provide the interphase between the “reading” and “writing” functions of signaling kinase cascades, where obviously, the writing function is the phosphorylation of specific tyrosine residues in the right downstream molecule [26]. The plasticity of the disordered regions may be further modulated by post-translational modifications and alternative splicing [27]. The former is favored by the availability of the disordered regions to modifying enzymes, while the second is enabled by the lack of structural constraints imposed by being part of a large, structured domain.

Not surprisingly, the Unique domain of Src has several phosphorylation sites that modulate its function. The PhosphoSitePlus database lists twelve experimentally confirmed serine/threonine phosphorylation sites in the Unique and SH4 domains of Src [28]. Serine 17, a substrate of PKA and related proteins [29], is preferentially phosphorylated in cancer cells [30]. Phosphorylation of serine-75, a substrate of various Mitogen-Activated Protein Kinases, causes changes in cell growth, cytoskeletal reorganization, and mediates ubiquitination and degradation of Src [31, 32]. Phosphorylation of threonine 37 activates Src by disrupting the interaction between the SH2 domain and regulatory phosphotyrosine 530 [33]. Phosphorylation of serine 43 and serine 51 by Wnt3A have opposite effects on Src activation [34].

### Non-canonical regulatory mechanisms. The role of the SH4 domain

The initial residues of Src family kinases form the SH4 domain, which mediates anchoring to membranes through myristoylation (attachment of the 14-carbon myristic acid to the N-terminus) and, in most members of the family, palmitoylation (attachment of the 16-carbon palmitic acid to the side chain of cysteine) [35]. Src is not palmitoylated, but its SH4 domain contains a cluster of basic residues that contribute to binding to negatively charged lipid membranes [36]. Membrane attachment is required for the transforming activity of the viral form of Src or the activation of Src by a membrane-bound phosphatase [37, 38].

In addition to the relatively well understood function of the SH4 domain in membrane attachment, the SH4 domain participates in other interactions that are likely to function as additional regulatory elements.

The SH4 and SH3 domains interact in the context of the fuzzy complex. NMR paramagnetic relaxation enhancement experiments that reveal the approximation of distant parts of a disordered region demonstrate that the Unique domain, in the absence of the SH3 domain, is preorganized to form the fuzzy complex but the SH4 domain is not. However, in the presence of the SH3 domain, the N-terminal SH4 region becomes an integral part of the fuzzy complex [22]. A co-evolution analysis using GREMLIN confirmed a functional interaction between the SH4 and SH3 domain [22, 39]. Furthermore, NMR confirmed that the SH3 domain contains a site to which the myristoyl group can bind when it is not inserted in a lipid membrane [40]. As a result, the strength of the interaction of Src with membranes can be modulated through the interaction of the SH4 and SH3 domains. Interestingly, in myristoylated Src, the Unique Lipid Binding Region does not contribute to membrane binding but stabilizes the intramolecular interaction of the myristoyl group with the SH3 domain and, therefore, competes with, rather than enhances, membrane binding.

A recent study has revealed a previously undiscovered interaction of the SH4 domain and the catalytic domain of Src that reduces Src activity and membrane anchoring. However, these effects could be reversed by abrogating the interaction either by deleting the SH4 domain or by mutations in the catalytic domain [41].

The possible interaction of the myristoyl group with the catalytic domain of Src has been suggested based on sequence similarity with the known myristoyl binding site in Abl [42, 43]. NMR studies confirm the interaction of a myristoyl group with an open conformation of Src [44]. This idea has been experimentally tested by the groups of Resh [45] and Moasser [46] through mutations affecting the Abl-like region of Src. The former group concluded that although a myristoyl binding site could be introduced by mutation, there was no evidence of an active myristoyl binding site in Src. On the contrary, Moasser suggests that the interaction of the myristoyl group of one Src molecule with the catalytic domain of a second molecule may be responsible for the observed Src dimerization detected by co-immunoprecipitation of tagged Src variants [46].

**Figure 2:**
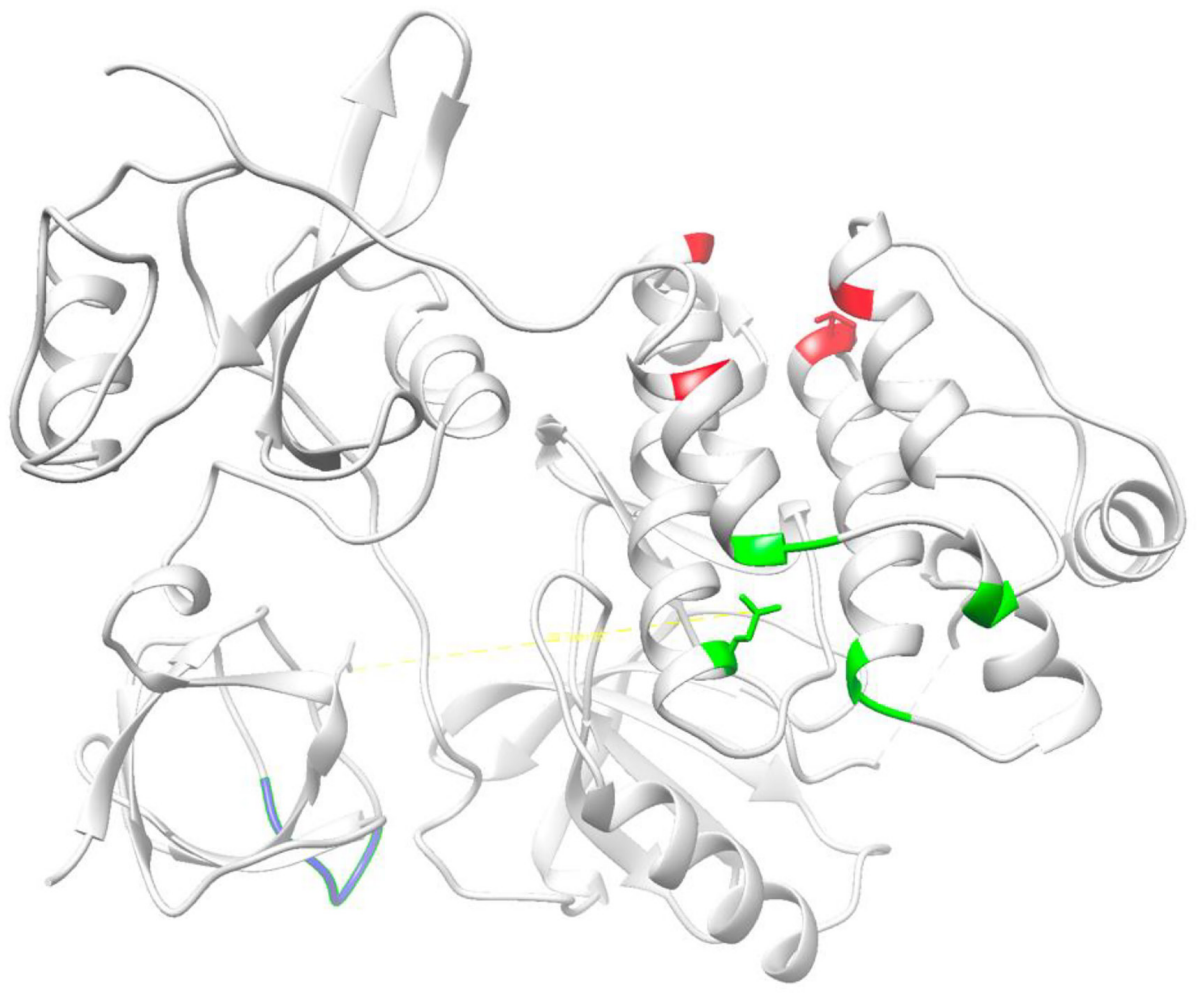
Proposed binding sites for the myristoylated N-terminal SH4 domain on the globular domains of Src based on experimental evidence provided by Ahler et al. [41] (red); Spassov et al. [46] (green); and Le Roux et al. [22, 40] (blue).

Interestingly, the interaction of the myristoylated SH4 domain with the globular region of Src has been independently reported by several groups, although the regions involved are quite distant in the X-ray structure of autoinhibited Src (Figure 2). The experimental evidence for the three proposals is strong and suggests a complex regulatory system in which multiple interaction sites either compete or cooperate modulating the conformational space of the active Src molecule in which flexible linkers connect the individual domains. The disordered Unique domain could enable the myristoylated SH4 domain to reach each of the individual sites.

### Non-canonical regulatory mechanisms. Src dimerization

Although it is known that c-Src activation involves trans-phosphorylation by a second c-Src molecule [47], membrane anchored c-Src has been generally assumed to remain monomeric. Nonetheless, Src dimers have been recently described by us and others [46, 48–50]. Moasser et al. suggest a model in which dimerization involves the interaction of the myristoyl group of one Src molecule with the SH1 domain of a second molecule that is bound to the inner side of the cytoplasmic membrane [46]. In the model systems studied by us, dimerization occurs in the absence of the SH1 domain but depends on membrane anchoring. It can be detected using surface plasmon resonance [49] or single molecule fluorescence [48] as persistent binding in model systems in which other effects, such as binding to lipid-rafts, can be ruled out. Surprisingly, Src dimerization on the membrane surface requires the cluster of lysine residues in the SH4 domain, in spite of the strong electrostatic repulsion [51].

### Non-canonical regulatory mechanisms. Phosphotransferase-independent Src functions

Src is a multivalent molecule that can act as a hub for multiple interactions, which may imply or not subsequent phosphorylation by the catalytic domain. Numerous examples of kinases have evolved functions beyond catalysis [52]. An extreme case are pseudokinases that have lost entirely their catalytic activity but are important actors in signaling pathways [53, 54].

Integrin signaling involves transient changes in Src kinase activity and phosphorylation of focal adhesion kinase (FAK). Defects in fibroblast spreading associated with deficient activation of Src by integrins could be complemented by either wild type or kinase defective Src, indicating that this phenotype does not require Src catalytic activity [55]. Similarly, fibronectin-stimulated signaling from a FAK-Src complex does not depend on the kinase activity of Src [56]. Osteopetrosis, resulting from defective osteoclasts in Src −/− mice can be partially rescued by the expression of kinase-inactive Src [57]. A recent example combining catalytic and non-catalytic roles of Src is the paradoxical activation of Src as a drug resistance mechanism, which implied the increased capacity of drug inactivated Src to bind to FAK, resulting drug-inactivated in enhanced activity of Src when the inhibitor concentration was reduced [58].

The role of Src, and other Src family kinases as interaction hubs is not restricted to integrin signaling. Other examples include Jak2 signaling induced by prolactin [59] and, even more surprising, the participation of Src in the formation of a complex between viral proteins NSP5A and NSP5B required for the replication of the hepatitis C virus [60].

The SH3 and SH2 domains have the capacity to mediate protein-protein interactions by interaction with proline-rich and phosphotyrosine residues, respectively, and their contribution to Src interaction hubs is not surprising. However, the Unique domain can also mediate protein-protein interactions. The most well-studied example is the anchoring of Src to synaptic NMDA receptors through the NADH dehydrogenase subunit 2 (ND2) that interacts directly with the Unique domain of Src [61]. This interaction can be targeted with cell-penetrating peptides based on the sequence of the Src Unique domain to treat chronic pain and hypersensitivity [62].

### Environmental sensitive signaling. Opposing roles of RA in the Src-YAP-IL6 pathways

**Figure 3:**
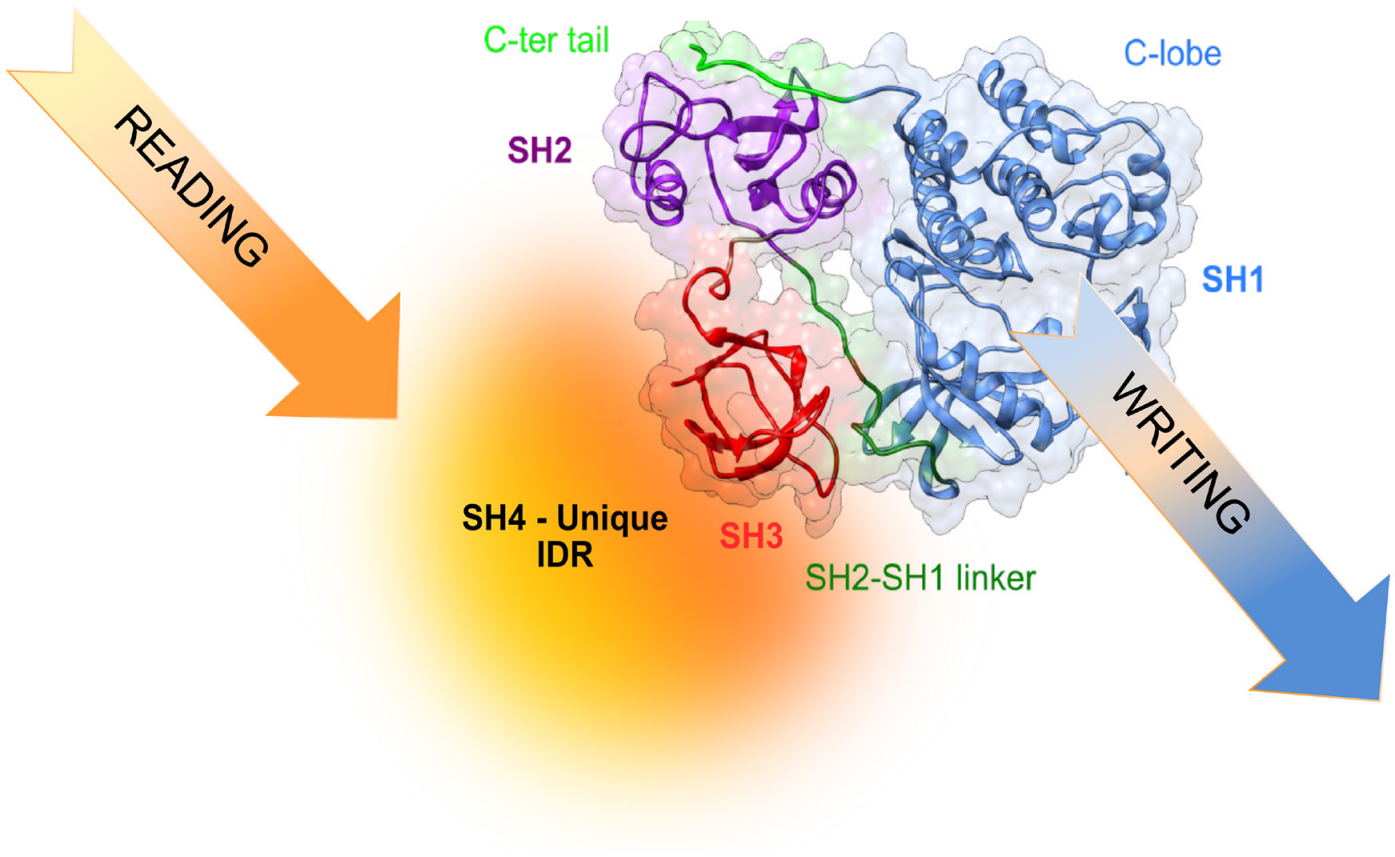
Translating information from the environment to generate cell-type selective signaling may involve reading of environmental signals by the disordered region and, eventually, modulating the activity and specificity of the kinase.

The capacity of intrinsically disordered regions of proteins to exist as an ensemble of rapidly interconverting forms offers a potent regulatory mechanism. The cellular environment can modulate the population of these interconverting forms by establishing weak interactions with other cellular components, or by reversible post-translational modifications (e.g., phosphorylation, acetylation, glycosylation). This would give a possibility to fine-tune the activity of proteins depending on the cell type and the cell environment (Figure 3).

The co-activator of transcription TIF2 was recently revealed to interact with retinoic acid receptors (RAR and RXR) through an intrinsically disordered region [63].

Retinoic acid (RA) is a molecule derived from the environmental factor vitamin A. Although RA has been effectively used in treating acute promyelocytic leukemia, it is already known that it can produce opposite effects in closely related subtypes of cancer cell lines. The effect of RA on triple-negative breast cancer cells derived from African American women (MDA-MB-468 cells) was tumor suppressive, while the effect on triple-negative breast cancer cells derived from European American women (MDA-MB-231 cells) was tumor-promoting, both *in vitro* and *in vivo* [64]. In a recent study we demonstrated that RA activated the pro-invasive and stemness-promoting Src-YAP-Interleukine-6 axis in MDA-MB-231 cells while it inhibited this pathway in MDA-MB-468 cells [65, 66].

### Environmental sensitive signaling. Src and stemness

The Src-YAP-IL6 axis controls invasion, metastasis, resistance to therapy, and stemness of MDA-MB-231 breast cancer cells [67]. This pathway is also overactivated in colon cancer [68, 69]. IL-6 is the first universal transcriptional target of YAP involved in promoting stemness conserved from flies to humans [67, 70]. Overexpression of IL-6 induces cancer cell proliferation, angiogenesis, and metastasis through stimulating STAT3, MAPK, and Akt signaling pathways. In addition, IL-6 regulates cancer stem cells, mesenchymal stem cell formation, and epithelial to mesenchymal transition in cancer and contributes to chemoresistance [71].

Other signaling pathways activated by Src include the Ras-Raf-MAPK-Erk2 and the PI3K-Akt-mTOR pathways, which are critical to transformation of chicken embryo fibroblasts [72]. These two pathways converge at several different sites within the cellular signaling network. For example, both pathways are involved in controlling the expression of c-Myc: Raf controls c-Myc expression at the transcriptional level [73]; mTOR controls c-Myc expression at the translational level [74]. Additionally, the expression of c-Myc is related to cancer stemness and drug resistance [75].

### Environmental sensitive signaling. Src and Yes in cancer resistance

Multiple signaling pathways activated through the activation of Src are related with the acquisition of drug resistance in cancer treatment [67, 71, 75, 76]. In relation to cancer resistance, Src is the most studied member of the Src family kinases (SFK), but increasing attention is being paid to Yes. YES1 is the only member of the SFK to show gene amplification in primary tumors of untreated patients [77] and as part of the development of resistance to chemotherapy and immunotherapy [78–81]. In addition, enhanced therapeutic anti-cancer response has been achieved by combining immune checkpoint and tyrosine kinase inhibition [82].

### Concluding remarks. Src as an oncotarget

Src is rarely mutated in human tumors, and overexpression of Src by itself in otherwise healthy cells is only weakly oncogenic [83]. Therefore, Src is probably not the lone dominant transforming factor in most cancers, but significantly contributes to cancer progression, resistance, and metastasis, and Src is overexpressed or hyperactivated in many human neoplasms, including colorectal, breast, pancreas, prostate, and lung as well as different types of sarcomas, glioma and melanoma. Thus, Src is a recognized oncotarget. Many small-molecule drugs have been developed and tested [76, 84–86]. Most drugs target the kinase or SH2 and SH3 canonical regulatory domains. While *in vitro* data are promising, the results from clinical trials have often fallen short of expectations. Possible reasons are lack of selectivity, resulting in toxicity, or the inhibition of the many physiological functions of Src in healthy cells. Since Src oncological effects results from deregulation in the specific context of cancer cells, a promising approach is to target the disordered region that includes unique sequences for each of the SFK and has no homologous regions in other kinases. Also, if our hypothesis that the fuzzy complex in the SNRE is an important player in Src’s sensing of its environment, targeting this region could achieve a cell-type selectivity that could ideally discriminate between healthy and cancer cells. Targeting of proteins’ intrinsically disordered regions was identified as one of the 10 top emerging technologies in 2019 by the World Economic Forum and it has been proven in other disordered cancer targets [87]. Our group has designed a screening system to search chemical libraries for binders of the Src SNRE [88] and we are currently following up a promising lead.
